# Sustained positive behaviour change of wounded, injured and sick UK military following an adaptive adventure sports and health coaching recovery course

**DOI:** 10.1136/bmjmilitary-2021-001784

**Published:** 2021-12-08

**Authors:** Rebecca J Sutton, C W P Kay, J McKenna, M Kaiseler

**Affiliations:** Carnegie School of Sport, Leeds Beckett University, Leeds, UK

**Keywords:** mental health, rehabilitation medicine, social medicine, sports medicine, adult psychiatry

## Abstract

**Introduction:**

A rising trend has occurred in the physical and mental health challenges faced by recovering UK service personnel. To support these individuals, bespoke inclusive multiactivity and adventurous training courses (MAC) have been developed. This study investigated the MAC’s influence on participants’ ability to sustain day-to-day changes that facilitate positive mental health and psychological need satisfaction.

**Methods:**

The 146 UK service personnel who participated in this study attended a five-day MAC 12 months ago. To investigate how the supportive experience influenced participants’ lives, quantitative and qualitative data were collected via an online survey. Open-ended questioning and abductive analysis were conducted to understand mechanisms, influential aspects of the course and positive behaviour change.

**Results:**

Positive behaviour changes were reported by 74% of the respondents. These changes align with positive psychological well-being (98%). Impactful elements of the course experienced by participants mostly aligned with the three basic psychological needs of autonomy (34%), competence (36%) and relatedness (61%).

**Conclusions:**

Recovery support programmes that encompass health coaching adventurous activities, such as the MAC, can initiate long-term positive behaviour change for recovering military personnel. In this specific context, the concurrence of the self-determination theory concepts that underpin the course delivery and participant outcomes is a powerful endorsement of implementation fidelity.

**Key messages**

- Unlike many outdoor adventure therapy programmes, the multiactivity and adventurous training course (MAC) is delivered to recovering UK military personnel who are still serving in the armed forces.
- The MAC is the only mandatory adaptive sports and adventure training course for UK Army and Royal Air Force (RAF) personnel in recovery.
- This health coaching and adaptive adventure sports course facilitates positive long-term behaviour change for recovering UK service personnel who took part in the research.
- Lessons from this research may be useful to other nations considering or developing their use of health coaching and adaptive adventure sports in supporting the recovery of their military personnel.
- This subclinical delivery style can complement the existing provision participants receive as part of their individual recovery plan.

## Introduction

UK service personnel have faced several intense and enduring conflicts in the last three decades, such as the Gulf, Iraq and Afghanistan wars.^1^ Sustained combat exposure has inflicted casualty rates as high as those suffered in the First World War.^2^ Consequently, the UK Armed Forces have experienced a rise in physical and mental health problems since 2012.^3^

A recent meta-analysis on the health impacts of military service found at least 67 000 UK veterans are likely to suffer from a physical or mental health condition.^1^ If wounded, injured or sick (WIS) personnel are unable to make a sufficient recovery and return to service, they may face medical discharge. The percentage of WIS personnel who went on to leave the services between April 2016 and 2017 was 72% in the RAF, 62% in the Army and 7% in the Royal Navy.^4^ Regardless of the final outcome, the uncertainty of injury and diagnosis of a career-impacting illness affects an individual’s long-term functioning and well-being, as well as military effectiveness and investment.^5^ Accordingly the Ministry of Defence recently reported the increased risk of occupational psychological injury among the UK service personnel, with prevalence of probable post-traumatic stress disorder and alcohol misuse at 6.2% and 10.0%, respectively,^6^ compared with a rate of 4% in the UK population.^7^ The Ministry of Defence has also suggested that serving military personnel are more likely to experience symptoms of common mental disorders than the general population.^6^

Changing behaviour is central to non-clinical interventions for recovering WIS personnel and there has been a shift in focus to non-clinical, behaviourally based support programmes to complement clinical care, with prioritisation of early intervention over treatment.^5 8 9^ Early intervention with recovering military personnel can introduce behaviours that protect and/or enhance well-being and can facilitate a positive approach for the remainder of an individual’s recovery.^5^ Adventure sports programmes aimed at supporting recovering military personnel are becoming commonplace, especially in the USA. For example, one consists of a six-month hike in which staff facilitate purposeful change through peer support, gradual resocialisation and physical activity. Four key themes emerged from veterans’ accounts of their experiences: improved social reconnection, life-improving change, inner peace and psychological healing, processing and reflection.^10^ Several similar studies have now shown outdoor adventure programmes to produce positive effects on well-being.^8 11–16^ While these outcomes are encouraging, the long-term impact of these programmes requires more indepth research into the transfer of the initiated positive behaviour change to the participants’ everyday lives. The multiactivity and adventurous training course (MAC) at the Royal British Legion’s Battle Back Centre is pioneering this approach within the UK Armed Forces. It is the first of its kind in the UK to support WIS serving personnel.

The MAC applies a strengths-based approach to human development.^17^ It provides a supportive adjunct to traditional clinical care by focusing on daily behaviours that activate personal potential and mental flourishing.^11 18^ The adapted sports and adventurous activities provide opportunities for restoration and development of cognitive, physical and social abilities through experiential learning.^11 12 19^ These activities may strengthen other recovery support processes participants are engaged in.^18^

The MAC is a mandatory requirement in the recovery pathway of all UK Army and RAF WIS and is available for recovering Navy personnel. It is the only mandatory adaptive sports and adventurous activity programme for recovering inservice Army and RAF military personnel. To be categorised as wounded, participants should have sustained battlefield casualties; injured are non-battlefield casualties, while sick are those living with systemic illness or mental ill health. The MAC is a five-day residential course that includes adaptive sports and adventurous training to facilitate personal growth and development. Throughout, the MAC adopts a participant-centred approach, underpinned by the self-determination theory (SDT), meeting the three basic psychological needs (autonomy, relatedness and competence) required to facilitate optimal psychological well-being and functioning.^20^ Autonomy relates to behaving with a sense of volition and the need to feel in control of one’s life and future. Competence encompasses mastering one’s environment. Relatedness represents the need to have positive relationships with others, oneself and one’s environment and harbour a sense of belonging and importance to others. The MAC supports participants in achieving their best possible recovery to return to duty or make a smooth transition to civilian life and facilitates long-lasting effects.^14^

There is substantial research evidence of the immediate positive influence of the MAC in participants.^13 16 21^ Between 2012 and 2015, 971 participants showed an average increase of 15.9% in positive mental well-being over the five-day recovery course.^13^ Addressing effects lasting beyond the immediate programme, this positive effect on mental well-being has increased to 33% in 759 participants in 2017–2018.^22^ Importantly, MAC participants also report a more rapid achievement of mental well-being and self-determination outcomes than from similar interventions delivered on different supportive programmes.^13 18^

A recent study investigating the long term impacts of the MAC on participants’ well-being after six months of completion^14^ has found that 75% of the 97 respondents evaluated the MAC as useful in facilitating adaptive changes. Additionally, relevant aspects of the course reported were aligned with the satisfaction of the three basic psychological needs of autonomy, competence and relatedness. Behaviours initiated and maintained six months later mostly aligned with improved psychological well-being, evidencing the success of the MAC in achieving its aims for UK service personnel recovery. The only exception was a single response out of 68 coded responses in which the participant explained that although they have tried they *“can’t find any happiness in myself or life.”* This response was coded as no change to daily life postcourse.^14^ Although this evidence is useful to shed light on the impact of sports courses on UK military personnel recovery and well-being after six months, the long-term sustainability of improvements and behaviour beyond six months postcourse remain unknown.^14 22^ This is a central area of investigation acknowledging the fact that reintegration in a civilian society, individual functioning and well-being are well-known challenges for the military personnel.^14 22^ The current study aims to extend this body of research by exploring the long-term impact of the MAC on the mental well-being of inservice, recovering military personnel 12 months following course completion.

### Aims of the study

- Understand the long-term impact of the MAC in recovering UK service personnel.
- Explore participants’ changed behaviours through the lens of positive psychological well-being (PPWB) 12 months post-MAC.
- Establish if the MAC has a sustained positive impact on the mental well-being and behaviours of participants.

## Methodology

### Study design

We conducted a qualitative exploration of the behaviour change of UK service personnel 12 months after attending the MAC. Participants completed the MAC between November 2016 and November 2018 and completed an online survey at a follow-up period of 12 months. The present study analysed the responses to one closed and two open-ended questions from the completed online survey to explore the experiences of UK service personnel 12 months after attending. The closed question asked: *‘Since being at The Battle Back Centre, have you made any changes in your day-to-day life?’* Participants could select either *‘Yes’* or *‘No’*. This was followed by the first open question which asked: *‘If yes, what changes have you made? Use the example sentence “I have stopped…” to help’*. The second open-ended survey question asked: *‘What part of the MAC had the greatest impact on you?’* This aimed to understand which aspects of the course were most influential for the participants.

### Procedures and participants

All participants completed the MAC and were invited to complete a 12-month follow-up survey between 7 November 2016 and 30 November 2018. Of the 579 participants who took part in the initial research during the course, 25% participated in the follow-up study. Only participants who answered all the questions were included in this study; this was 87 participants, 15% of all those who were contacted and invited to take part in the longitudinal study.

From the 87 participants who provided full responses, 81% were male and 19% were female, ranging in age from 20 to 52 years (mean=33.75 years). At the time of providing follow-up information for the present study, the participants may have returned to duty, by completing a recovery programme, or have been medically discharged from the armed forces. The distribution of participants in each category is not known as this was not included within the ethical approval secured for evaluation research of the MAC experiences during follow-up. Regardless of their subsequent military status, all participants had provided consent for the evaluative research. A participant information sheet and a consent form were provided to all MAC participants at least 24 hours prior to attendance; written consent was obtained on arrival. Continued consent (online) was confirmed by participants prior to completion of the 12-month follow-up survey.

### Overview of the study intervention

The five-day MAC is based on adaptive sports and adventurous training to facilitate recovery. Each day the MAC commences with an educational session led by specialist coaching staff. This introduces psychological concepts and coping strategies, for example, motivation, attitude and goal setting, which aid recovery. Teaching content is then contextualised each day by a person-centred delivery approach underpinned by the SDT and daily opportunities to develop the three psychological needs: autonomy, competence and relatedness.^23^ Regular training is provided to all delivery staff for using these SDT concepts; process evaluations confirmed that these concepts feature in all sessions.

Daytime activities begin with short discussions around adaptive thought processing and mental resilience. The day is then filled with various adaptive sports and adventurous training, including kayaking, mountain biking, wheelchair basketball, archery, caving and climbing, delivered through a *‘challenge by choice’* approach. This enhances participants’ autonomy; they choose their level of engagement.^14^ Autonomy is also facilitated by opportunities to master sports-based challenges. An extensive array of bespoke adaptive equipment, supported by a technical advisor, ensures complete participation through activity adaptation to suit the needs of any individual. Daily reflections are facilitated to extend participants’ understanding of personal development and behaviour change. Based on small and whole group discussions, the experiences of the day’s activities are reviewed; this peer-to-peer approach further ensures the experience of relatedness.^24^

### Data analysis

Qualitative responses to the open-ended questions were analysed using content analyses. An abductive approach was adopted to code responses using preidentified codes: health behaviours (smoking, alcohol consumption, sleep, physical activity/occupational activity, food consumption) and PPWB (positive emotion, optimism and life satisfaction) for the first open-ended question. The second open question was analysed using the preidentified codes of SDT: autonomy, competence and relatedness.^25^

Existing research analysing participants’ experiences of the MAC at a six-month follow-up investigated the *‘started’* changes associated with restorative behaviours.^14^ The current study also investigated *‘stopped changes’* with a focus on ending unhelpful and/or destructive behaviours.^15 25^ Shanahan *et al* 15 identified that either a change to the environment or a change in behaviour is needed for any intervention to improve health and well-being. The behaviours positively correlated with PPWB were used as preidentified codes to analyse the qualitative responses to the second open question.

## Results

Of the 146 UK service personnel who voluntarily completed the 12-month follow-up online survey, 118 responses were provided to the closed and two open-ended questions. The question *‘Since being at The Battle Back Centre, have you made any changes in your day-to-day life?’* received 87 (74%) answers for yes and 31 (26%) answers for no. In response to the open-ended question *‘If yes, what changes have you made? Use the example sentence “I have stopped…” to help’*, out of 87 answers provided some participants reported more than one change and therefore a total of 95 responses were coded. All 95 responses were coded in line with health behaviours: smoking, alcohol consumption, sleep, physical activity/occupational activity, food consumption and PPWB: positive emotion, optimism and life satisfaction.^25^ The majority of responses implied positive changes in behaviour, with only two responses reporting worsening changes (see Table 1).

**Table 1:** Main themes for the question *‘Since being at the Battle Back Centre have you made any changes in your day-to-day life? If yes, what changes have you made? I have stopped…’* (N=87)

Theme	No/negative change example quotes	Positive change example quotes	Frequency (n)
Smoking	–	“Smoking.” “Quit smoking.”	5
Alcohol consumption	–	“Stopped drinking all the time.” “Drinking as much.” “Alcohol consumption in great volumes.” “Drinking alcohol.” “Cut down on the drinking.”	12
Sleep	–	“Staying up really late.” “Sleeping as often as I used to.”	4
Physical/occupational activity	“Physical activity – negative change.”	“No longer sofa bound.” “Thinking I can’t do something that is, I can’t go out.” “Staying at home all the time.”	7
Food/diet	–	“Taking sugar in tea and coffee.” “Feeling so tired all the time because I am not so unwell because I became Vegan.”	2
Positive emotion	“Unfortunately, I have been feeling low again and struggling.”	“Stopped feeling so negative.” “Bottling up thoughts and feelings.” “Being so hard on myself.” “Shouting, much calmer.” “Negative self-talk.” “Excessively stressing.” “Feeling sorry for myself.”	27
Optimism	–	“Stopped thinking I can’t do something, because I can at least try.” “Doubting myself and permitting a negative mindset.” “Putting myself down at every opportunity.” “Thinking I am unable to do this.” “Thinking that the worst will happen.” “Being totally negative.” “Assuming my own limits.”	17
Life satisfaction	–	“Stopped putting others before myself all the time and stopped taking work home.” “Thinking that everyone in the Military treats you bad.” “Thinking about the past as much.” “Beating myself up if I don’t get 101 things done in a day – doing fewer things better.”	21

Of the day-to-day changes reported by the participants, 98% positively correlate with PPWB. In relation to the second open-ended survey question *‘What part of the MAC had the greatest impact on you?’*, out of 87 responses some participants reported more than one element of the course, providing 116 responses to be coded. The 116 responses have been coded in alignment with the three basic psychological needs of autonomy, competence and relatedness that were experienced during participation in the MAC activities (see Table 2).

**Table 2:** Main themes for the question *‘What part of the multiactivity course had the greatest impact on you?’* (N=87)

Theme	Elements of the course example quotes	Frequency, n (%)
Autonomy	“Realising more about myself and that I am in charge of my own destiny and that all the things I do are based on choices.” “The ability to stop and take stock and have time for reflection.” “Coping mechanisms.” “The instructors, they were the catalyst for me to understand I was worthwhile, I could face life and I could overcome. They saved my life, gave me self-belief to go on living.” “The coaching to work through situations that make me uncomfortable and to work on my strengths.” “Coming away with tools to help me move forward.” “Made me realise that I need to get the balance of energy input and output right on a regular basis and find people and activities that help restore my reserves.” “Having a sense of purpose again.”	32 (34)
Competence	“Learning I can do things if I put my mind to it.” “Overcoming challenges such as being afraid of heights and having the confidence to get to the top [of the climbing wall].” “Being able to help others.” “Being reasonably successful in some activities.” “Rock climbing was very much a big help to me.” “Getting motivation to do tasks.” “Things like basketball because it reminded me that it’s okay to have fun and smile.” “Getting into a pool and overcoming my fear of water, since then I’ve completed an intense swimming course and paddled the Great Glen.” “Facing some challenges which I was initially apprehensive about, but which proved to be positive experiences.”	31 (36)
Relatedness	“Meeting like-minded people.” “The opportunity to meet others in a similar situation to me.” “The camaraderie.” “Getting to meet other people who are going through the same things that I am going through in my own life.” “Talking candidly amongst peers, having conversations that usually do not happen.” “The others around me, who were on the same journey to civilian life.”	53 (61)

Responses could be coded as more than one theme.

## Discussion

This study extends existing adaptive sports and adventure training research and offers a novel insight into the efficacy of the MAC in initiating enduring impacts for participants. The results suggest the MAC facilitated the three basic psychological need satisfaction and PPWB for the UK military personnel 12 months after attending the MAC.

These changes are related to undertaking positive behaviours; consistent with expectations, this was associated with improved psychological well-being. Activities and coaching aspects of the course satisfied the three basic psychological needs of SDT, of which underpins the MAC design and delivery: autonomy, competence and relatedness.^20 25^ The most prominent change reported in relation to PPWB was positive emotion, followed by life satisfaction and optimism. Restorative behaviours and positive behaviour change (ie, reduction in alcohol consumption and smoking) were reported by 35% of the participants.

The positive impact of the MAC is evident in these findings and suggesting that near transfer is facilitated from activities during the course to everyday life challenges.^14^ Participants stated *“[I] realised more about myself and that I am in charge of my own destiny and that all the things I do are based on choices”* and *“[Learnt] I can do things if I put my mind to it”*, which imply that the programme facilitated the development of participants’ positive mindsets, and improved self-confidence and purpose in life and the ability to manage behaviours. These statements indicate that the MAC activated an adaptive level of *‘perspective-taking’* that further activated positive behaviour change.

Unhelpful behaviours such as negative mindsets, alcohol misuse and low life satisfaction were reported to have been stopped, allowing for an increase in self-acceptance and personal growth, in turn improving the quality of life. These findings may be explained by the experiential learning and person-centred approach of the MAC delivery by expert coaching staff. A recent study quantitatively explored the short-term impacts of an adapted sports recovery programme at the Battle Centre for UK service personnel and found statistically significant increases in positive mental health and basic psychological need satisfaction.^13^ These significant findings were also reported at a six-month follow-up conducted via qualitative abductive analysis, where 75% of the participants reported a positive change in daily behaviours six months following completion of the MAC.^14^ In line with these findings, the present research shows that the MAC continues to activate positive change and reduce negative behaviours at 12-month follow-up postcourse. This attends to a widely acknowledged gap in the research field for longitudinal and more vigorous research.^11^

Psychological well-being research has increasingly reported the wider implications of mental health and well-being in relation to physical functioning, brain resilience and social interactions.^17^ These positive outcomes are evidenced a year after attending the MAC.

By framing the design and delivery around the SDT and its three basic psychological needs, this study extends previous research showing the short-term impacts of the MAC.^13 14 16^ Now we can see benefits, consistent with the short-term enhancements, at 12 months.

These findings should be considered with acknowledgement of the study’s limitations to inform future research. This work assessed self-reported survey responses by participants and adopted an exploratory qualitative analysis; this process has a limit to the depth and breadth of information that can be collated. With only 87 fully completing the survey out of the 146 who participated, this meant some participants’ responses were omitted in order to obtain reliable and consistent data; the information provided evidence of respondents’ results and is not implied to represent all participants’ experiences of the MAC after a follow-up period of a year.

Future research should consider more comprehensive qualitative methods less reliant on self-reported measures, for example, a mixed methods study design using interviews and self-reported questionnaires or daily diaries to more fully understand the psychological well-being experiences of participants a year after the MAC. Long-term impact research should also consider comparing data collected at more regular intervals to explore any external influences which may have impacted on participants’ well-being. Future research is also needed to explore the long-term impacts of similar adventure sports interventions among serving military personnel to compare outcomes and better understand the design and delivery of successful mental well-being recovery programmes.^18^ Analyses of course components, for example, nature, warrant more indepth consideration considering the importance that previous literature has identified surrounding such variables.^19^ By addressing the limitation of the present study and blending the strengths of existing literature and what is now known regarding the success of MAC among UK WIS MP, subsequent research can focus on refining and defining these findings even further.

To conclude, the present study offers a novel contribution and significant extension to existing literature by highlighting the enduring impacts of adaptive sports and adventure training programmes in activating UK WIS military personnel to make constructive changes in everyday life. The MAC was also linked to reducing harmful behaviours. Studies showing the temporal patterns of change, post-MAC, are now needed to show the direct and indirect effects attributable to the MAC. This research also strengthens the support for use of SDT as an underpinning theoretical framework for adventure sports programme design to facilitate long-term improvements in health behaviours and PPWB.^15^ The utility of perspective-taking, developed through the reflective activities that surrounded each day of outdoor adventure activities during the MAC, should also be investigated. Psychological well-being research has suggested the potential facilitative nature of a person-centred approach with regard to mental health intervention outcomes; this is a key feature of the MAC delivery and may explain the positive impacts.

## Data Availability

Data are available upon reasonable request.
